# Important Role of the IL-32 Inflammatory Network in the Host Response against Viral Infection

**DOI:** 10.3390/v7062762

**Published:** 2015-06-16

**Authors:** Yaqin Zhou, Ying Zhu

**Affiliations:** State Key Laboratory of Virology, College of Life Sciences, Wuhan University, Wuhan 430072, China; E-Mail: zhouyq1993@whu.edu.cn

**Keywords:** interleukin-32, pro-inflammatory cytokine, virus infection, antiviral activities

## Abstract

The pro-inflammatory cytokine interleukin (IL)-32 has gained much attention recently because of its important role in the inflammatory network. Since the discovery of IL-32 in 2005, our appreciation for its diverse roles continues to grow. Recent studies have discovered the antiviral effects induced by IL-32 and its associated regulatory mechanisms. The interactions between IL-32 and various cytokines including cyclooxygenase 2 (COX-2), inducible nitric oxide synthase (iNOS), interferon (IFN)-λ1, interleukin (IL)-6, and soluble IL-6 receptor have been described. This review aims to integrate these new findings into explicit concepts and raises the intriguing possibility of IL-32 as a therapeutic target.

## 1. Introduction

Interleukin (IL)-32, known as a pro-inflammatory cytokine, has gained much attention recently because of its important biological functions. It is mainly expressed by natural killer cells, T cells, epithelial cells, and blood monocytes [1]. Interestingly, IL-32 was called “natural killer cell transcript 4 (NK4)” when first identified in 1992, because it was found to be selectively expressed in activated T cells or natural killer cells [2]. The biological function of IL-32 remained undiscovered until 2005 [3]. In that report, Kim *et al.* demonstrated that recombinant NK4 could induce several pro-inflammatory cytokines, including tumor necrosis factor α (TNF-α) and IL-8. Hence, NK4 was renamed IL-32. The IL-32 gene is located on the human chromosome 16p13.3 and exists as six splice variants: IL-32α, IL-32β, IL32γ, IL-32δ, IL-32ε, and IL-32ζ [4]. The respective biological activities of each splice variant have been described in published reports [5,6,7,8,9]. For example, IL-32β enhances the adhesion of immune cells to activated endothelial cells [10]. IL-32γ has antiviral effects against the influenza A virus (IAV), human immunodeficiency virus (HIV), herpes simplex virus 2 (HSV-2), and vesicular stomatitis virus (VSV) [11,12,13,14]. Among those isoforms, IL32γ is the most biologically active [15]. Nevertheless, the specific biological functions of each isoform are not yet understood. IL-32 has no similarities with other known cytokines, but exhibits the typical pro-inflammatory properties [3,16,17]. It is worth noting that the IL-32 gene has been identified in most mammals except for rodents; the lack of a mouse IL-32 gene limits *in vivo* studies and further development of IL-32 research for clinical applications [18]. IL-32 leads to the activation of the p38 mitogen-activated protein kinase (MAPK), nuclear factor κB (NF-κB), and activator protein-1 (AP-1) signaling pathways as previously reported [3,17,19,20]. IL-32 was found to stimulate the production of multiple chemokines and pro-inflammatory cytokines, including IL-1β, TNF-α, IL-6, IL-8, and macrophage inflammatory protein-2 (MIP-2) [3,17,19,20]. The expanding knowledge of IL-32 indicates that it plays a vital role in inflammation or infection with various pathogens. Mycobacterium tuberculosis, Epstein-Barr virus (EBV), HIV, hepatitis B virus (HBV), hepatitis C virus (HCV), and IAV all induce the expression of IL-32, as described [11,12,21,22,23]. Besides virus infection, IL-32 is involved in inflammatory diseases such as ulcerative colitis, vasculitis, rheumatoid arthritis, and Crohn’s disease [3]. In this review, we focus our discussion on the antiviral role of the pleiotropic cytokine IL-32.

## 2. IL-32 Crosstalk with Cytokines in the Host’s Antiviral Response

IL-32 can regulate or be regulated by several cytokines, including cyclooxygenase 2 (COX-2), inducible NO synthase (iNOS), interferon (IFN)-λ1, interleukin-6 (IL-6), and soluble IL-6 receptor. This “crosstalk” makes IL-32 a crucial mediator of the host’s antiviral response.

### 2.1. Cyclooxygenase-2 (COX-2)

Serum levels of IL-32 were measured and found to be significantly elevated in patients with IAV infection [1]. Likewise, IAV infection stimulates COX-2 expression, followed by the prostaglandin E2 (PGE2) accumulation in human lung A549 cells [1,24,25]; double-stranded (ds) RNA and NS1 (non-structural protein 1 of IAV) are the key viral components involved in this process [1]. Furthermore, the level of PGE2 can be measured to assess COX-2 activity [24,26,27,28]. COX-2 was shown to regulate dsRNA-triggered IL-32 production in A549 cells, which revealed that COX-2 is an upstream regulatory factor of IL-32. IL-32 promoter activity was increased by the over-expression of COX-2 and suppressed by the COX-2 inhibitor NS398. Concurrently, the over-expression of IL-32 inhibited the COX-2 biosynthesis, which revealed a negative feedback mechanism. Research showed that PGE2 production was suppressed by the IL-32 over-expression in a dose-dependent manner and increased by the IL-32 knock-down [1].

In another study, ionizing radiation (IR) dramatically induced IL-32 expression in vascular endothelial cells [29]. The production of the cytosolic phospholipid A2 (cPLA2) was also increased upon the exposure of endothelial cells to IR, which was followed by the release of free fatty acids, mainly arachidonic acid (AA). AA was further metabolized to prostaglandins depending on the presence of COX-2. Blocking of COX-2 expression decreased the IL-32 expression. Results from another study indicated that prostaglandin I2 (PGI2) plays a key role in the up-regulation of IR-induced IL-32 [29].

Activation of caspase-1 resulted in an increased IL-32 and thymic stromal lymphopoietin (TSLP) production [30,31]. Indeed, activated caspase-1 promoted COX-2-dependent inflammatory reactions, which further increased IL-32 production [32].

IL-32 expression was elevated in cervical cancer tissues and human papilloma virus (HPV)-positive cervical cancer cells [33]. The high-risk HPV-16 E7 oncogene was shown to be involved in this process, because the treatment of cells with E7 antisense attenuated both the IL-32 and COX-2 expression levels. Moreover, IL-32 expression was blocked by the COX-2 inhibitor NS398 and increased by the over-expression of COX-2. Nevertheless, E7 and COX-2 expression were decreased in the IL-32γ over-expressing cells, and this attenuation was recovered by IL-32 small interfering RNA (siRNA), which revealed a negative feedback mechanism among the three genes [34].

In conclusion, these findings are consistent with the published results, which state that COX-2 up-regulates IL-32 expression, while IL-32 feedback inhibits COX-2 expression.

COX-2 is the inducible form of cyclooxygenase. The induction of COX-2 by any type of inflammatory stimulus quickly results in the biosynthesis of prostaglandins of the E-series, mainly PGE2, which in turn orchestrates the inflammatory response [35]. Studies showed that aspirin and all the other nonsteroidal anti-inflammatory drugs (NSAIDs) exert their anti-inflammatory effects primarily through the inhibition of cyclooxygenase [35]. Previous research identified COX-2 as an obligatory mediator in airway inflammation during IAV infection [25]. In addition to IAV, the activation of COX-2 by EBV, HBV, HCV, herpes simplex virus type 1 (HSV-1), bovine ephemeral fever virus, and the classical swine fever virus indicates a significant role for COX-2 in the antiviral response [33,36,37,38,39,40,41]. Hence, the antiviral effects of IL-32 are not only directly involved in the inflammatory response, but are also indirectly involved in the regulation of COX-2.

### 2.2. Inducible NO Synthase (iNOS)

Viral infections, including IAV infection, increased IL-32 and inducible NO synthase (iNOS) expression [1,13,36,42]. DsRNA and NS1 were identified as responsible for IL-32 and iNOS activation. Furthermore, IL-32 treatment alone also significantly increased both NO production and iNOS expression [1,10,13,36,42]. Over-expression of IL-32 increased the iNOS promoter activity in various cell types, such as A549, Jurkat, U937, and HEK 293T cells. The stimulation of NO was suppressed by the IL-32-specific siRNA, which indicated that IL-32 is an upstream regulatory factor of IAV-triggered iNOS expression. iNOS over-expression suppressed the IL-32 promoter activity and mRNA levels in the A549, Jurkat, U937, and HEK 293T cells. In contrast, treatment with the selective iNOS inhibitor *S*-methylisothiourea sulfate enhanced IL-32 production. These results indicated that iNOS-derived NO exerts feedback inhibition on the IAV-induced IL-32 expression [36].

In another study, iNOS expression and NO production were increased by the IL-32 treatment in the lipopolysaccharide-stimulated rat primary astrocytes [10]. IL-32 up-regulated the mRNA expression of iNOS along with MMP-9, TNF-α, and TGF-β in rat primary astrocytes [10]. It was reported that IL-32 promoted extracellular signal-regulated kinase (ERK)1/2 phosphorylation [43,44,45], which further induced iNOS and matrix metalloproteinase 9 (MMP-9) expression in rat primary astrocytes [46]. These findings suggested that IL-32 mediates the inflammatory activation of astrocytes via the ERK1/2 pathway [10].

iNOS, also called NOS2, is present mainly in macrophages and neutrophils. It was reported that iNOS is localized in the nucleus, although its specific biological role is unknown [47,48,49,50]. Through a complex oxidoreductase reaction, iNOS converts l-arginine and oxygen into l-citrulline and NO. Another source of NO is nitrite obtained through an enzymatic reaction, or non-enzymatically under acidic conditions [47]. NO produced from iNOS is a significant pro-inflammatory mediator that has direct antiviral properties, but also can mediate immunopathology or inhibit the antiviral immune response to promote chronic infection [51]. Studies of the antimicrobial and antiviral activities of iNOS were performed mostly in macrophages, but the iNOS-dependent effector functions have been found in other myeloid or non-myeloid cells [47]. NO interacts directly with the components of the replication machinery, metabolic enzymes, structural elements, and the nucleic acids of infectious pathogens to exert antiviral or antimicrobial effects [52,53]. Therefore, NO inhibits pathogen proliferation, causes DNA mutagenesis, blocks metabolism, and inactivates the virulence factors of pathogens [47]. Indirect effects of iNOS against the many types of infectious pathogens have also been observed. For example, it was reported that iNOS-triggered NO production restricts intracellular M. tuberculosis growth via eliciting the apoptosis response in the host cell [54].

Increasing knowledge of iNOS and NO will provide a new perspective into the host response to infection. Because iNOS and NO have been shown to possess antiviral activity, the regulation loop of IL-32 with iNOS and NO offers new insight into the role of IL-32 in the antiviral response.

### 2.3. Interferon (IFN)-λ1

Elevated IL-32 expression and suppressed microRNA-29b levels induced by HBV have been observed in various cell types [22]. Remarkably, the treatment with human hepatitis B immunoglobulin (the HBV-neutralizing antibody) suppressed the increased expression of IL-32. NF-кB is an important *cis*-regulatory element within the IL-32 promoter [12]. HBV-induced IL-32 promoter activity could be dramatically impaired by the deletion or mutation of NF-κB binding sites. Indeed, the binding of NF-κB p65 to the two specific regions within the IL-32 promoter was enhanced by HBV [22]. Therefore, it was concluded that HBV stimulated IL-32 transcription mainly through the activation of the transcriptional factor NF-κB. In addition, the suppression of microRNA-29b contributed to the HBV-induced IL-32 activation [22]. Coincidently, a weak inverse correlation between microRNA-29b and IL-32nonα levels in PBMC collected from HIV-1-infected patients was observed, indicating a role of microRNA-29b in antiviral response [55]. In HBV patients, IL-32γ and IFN-λ1 levels were increased and positively correlated with each other. IL-32 effectively inhibited the expression of hepatitis B s and e antigens, and the HBV intracellular core protein in the peripheral blood mononuclear cell (PBMCs). However, the anti-IFN-λ1 antibody attenuated the antiviral function of IL-32 [22]. Further study showed that the inhibitory function of IL-32 was dependent on IFN-λ1. Knock-down of IL-32 by the specific short hairpin RNA (shRNA) inhibited IFN-λ1 expression. IL-32 activated NF-κB to stimulate the expression and secretion of IFN-λ1, which has universal antiviral activities [22]. Other studies have reported that the treatment with IL-32 upregulated PKR, MxA, and APOBEC3G/3F levels in different cells [14,56]. These interferon stimulated genes (ISGs) are regulated by type I IFNs as well as IFN-λ subtypes which share the same JAK–STAT signaling pathway for the activation of the antiviral response with the former. The relationship between IL-32 and these ISGs also indirectly reflect the antiviral ability of IL-32 depending on IFNs. Besides IFN-λ1, the interaction between IFN-γ and IL-32 has also been reported. IFN-γ pretreatment sensitizes human umbilical vein endothelial cells and PBMCs to exogenous IL-32γ and dramatically increases IL-6 and TNF levels [57]. It suggests that IFN-γ may work as a cofactor for IL-32 to exert its biological function.

IFNs play a very important role in antiviral protection because they are the key cytokines that mediate an extensive antiviral response. IFN-λ1, also known as IL-29, belongs to the type III IFNs, which include IFN-λ1 (IL-29), IFN-λ2 (IL-28A), and IFN-λ3 (IL-28B). Compared with type I IFNs, type III IFNs exhibit differences in the structure, genetics, and receptors, but similarities in the mechanisms of induction, signal transduction and biological activities [58]. After viral infection, interferon regulatory factors (IRF) 3, IRF7, and NF-κB are activated, which induces the production of type III IFNs [59]. Research showed that type III IFNs induce the activation of the JAK/STAT signaling pathway and IFN-stimulated response element expression [60]. HBV and HCV replication were shown to be impaired by type III IFNs [61]. IAV and Sendai virus-triggered type III IFN production has also been described [62]. In addition to the viruses discussed above, several other viruses have been shown to induce the expression of type III IFNs, including the encephalomyocarditis virus, the human and murine cytomegaloviruses, the Apeu virus, the Sindbis virus, and HSV-2 [61,62,63,64,65,66,67]. The activation of IFN-λ1 endows IL-32 with extensive antiviral properties, which explains the key role of IL-32 in the viral infection response from yet another perspective.

### 2.4. Soluble Interleukin-6 Receptor (sIL-6R) and IL-6

The expression of soluble IL-6 receptor (sIL-6R) was found to be significantly higher in IAV- and HBV-infected patients than in healthy individuals [68]. The COX-2 pathway regulates sIL-6R expression in IAV-infected cells, which is independent of IL-6 [68,69]. Further study indicated that IAV up-regulated the sIL-6R expression not only in patients, but also in A549 cells, PBMCs, and the MRC-5 human embryonic lung diploid fibroblast cell line [68]. Increased sIL-6R production was followed by the IL-6, IL-10, IL-21 and IL-32 promoter activation. However, this effect was reversed after the cells were treated with sIL-6R shRNA; the suppression of IL-32 and IL-6 expression was particularly remarkable. The over-expression of sIL-6R led to the enhanced IL-6 mRNA and protein levels, while the knock-down of sIL-6R suppressed IL-6 production in IAV-infected cells. A similar result was observed with IL-32 expression. Hence, sIL-6R was shown to be an upstream regulator of IAV-induced IL-32 and IL-6 activation, and the regulation of sIL-6R expression by IL-32 appears to follow a feedback mechanism during IAV infection. The mRNA and protein levels of sIL-6R were dramatically reduced in A549 cells when treated with excess IL-32. In contrast, IL-32-specific shRNA treatment resulted in a dose-dependent increase of sIL-6R. It was noted that IL-6 had no apparent effect on the sIL-6R stimulation [68]. IL-32 stimulated IL-6 production in PBMCs and A549 cells infected with IAV, while IL-32 knock-down significantly reduced IL-6 expression [17,68]. In another report, IL-32 serum levels increased prominently in H1N1 influenza A infected patients compared to normal healthy individuals, and it was found that the antiviral activity of IL-32 is via a THP-1 cell-produced factor, transferrin [70]. Interestingly, IL-6 induction was tightly associated with IL-32 level, suggesting that IL-6 induction in the patients depends on IL-32 level [70].

IL-6 was up-regulated by both sIL-6R and IL-32, as mentioned above. Indeed, it was shown that sIL-6R promoted IL-6 production via IL-32 [68]. Another report describes the specific mechanism of regulation via PKCε of PKC family [71]. PKC is a family of serine/threonine kinases [71], and PKCε is one of several isoforms shown to have positive effects on IL-6 expression [72,73]. Signal transducer and activator of transcription 3 (STAT3) can also promote IL-6 production in serum-starved cancer cells [71]. The levels of IL-6 mRNA in THP-1 promonocytic cells were markedly higher upon treatment with over-expressed IL-32α together with the PKC activator PMA than with IL-32α alone. The treatment of cells with Ro-31-8220, a PKCε inhibitor, completely abrogated the augmenting effect of IL-32α on IL-6 production, which indicated an important role of PKCε in the IL-32α-triggered IL-6 expression. Indeed, PMA treatment of THP-1 cells resulted in STAT3 (Ser-727) phosphorylation. Experimental data showed that the phosphorylation of STAT3 was modulated by IL-32α via PKCε, and that IL-32, PKCε, and STAT3 form a trimeric complex to regulate the IL-6 production [71].

IL-6 is an inflammatory cytokine produced mainly by T cells, macrophages, and adipocytes [69]. It has two types of receptors—the membrane-bound IL-6 receptor and the soluble IL-6 receptor. IL-6 has always been considered the prototypic pro-inflammatory cytokine that is involved in the pathogenesis of all inflammatory diseases [74]. The signal transduction of IL-6 is induced by the binding of IL-6 to its specific alpha receptor. There are two signaling pathways used by IL-6—the classic signaling pathway via the membrane-bound IL-6 receptor, and the trans-signaling via the sIL-6R. The latter is considered to mediate chronic inflammation and cancer development [74,75]. Through the combination of IL-6 and sIL-6R, the signaling complex can generally activate all the cells in the human body [74]. IL-6 is a pleiotropic cytokine, which plays a role in hematopoiesis, regeneration, and the progression and development of cancer [76]. The association between IL-6 and viral infection was partially revealed in published studies [77,78,79,80]. For example, in virus infections, the expression of IL-6 in follicular B cells of the draining lymph node induces the production of critical cytokines such as IL-21; this is a necessary early event during the antiviral response [81]. Studies have demonstrated that numerous viruses, including IAV, VSV, enterovirus 71 (EV71), and HBV induce sIL6R production via the COX-2 pathway [69]. sIL6R elicits extensive antiviral activity independent of IL-6; it induces type I IFN expression and activates the IFN downstream effectors through the p28 pathway [69].

In conclusion, sIL-6R acts as an upstream regulatory factor for IL-32 during viral infection and promotes IL-6 production via IL-32, while IL-32 feedback inhibits the IAV-induced sIL-6R expression [68]. The induction of IL-6, mediated by IL-32, is crucial for the host response against viral infection. The correlation between IL-32 and sIL-6R also exerts an important impact on the antiviral response. These findings shed new light on the antiviral properties of IL-32.

**Figure 1:**
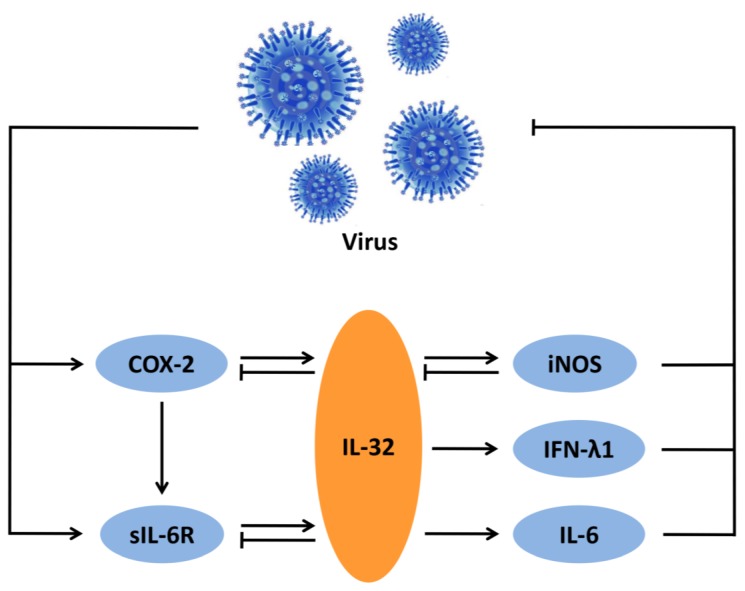
The IL-32-mediated inflammatory network against viral infection. Virus infection induces COX-2 and sIL-6R expression, which upregulates IL-32 production, resulting in increases of iNOS, IFN-λ1, and IL-6 levels. However, elevated IL-32 level feedback inhibits COX-2 and sIL-6R expression; iNOS also has a negative feedback effect on IL-32. This network plays a key role in host response to viral infection.

Together, a schematic diagram for the antiviral action of IL-32 is presented in Figure 1.

## 3. Conclusions

Since its initial description as a natural killer cell transcript in 1992 [2], our understanding of the biology of IL-32 has gradually increased. We can conclude from the literature that IL-32 is a critical pro-inflammatory mediator, which has essential antiviral functions. Whether activating iNOS, IFN-λ1, and IL-6, or inhibiting COX-2 or sIL-6R by a feedback mechanism, the IL-32-centered inflammatory network has become an important presence. Because IL-32 plays a key role in the inflammatory response against virus infection, developing a therapeutic strategy that targets IL-32 should be an important research field. Considering that IL-32 expression is increased during the infection with various viruses, the up-regulation of IL-32 might be a promising therapeutic strategy, and the identification of the specific biological function of each IL-32 isoform would be of great importance in this endeavor. One problem that remains unresolved is the identity of the cell surface IL-32 receptor, which directs the targeting of the IL-32 signal from the outside of the cell. There have been several studies focused on the extracellular activities of IL-32, which have indicated the potent effects on IL-32 by integrin signaling [82]. Despite this recent progress, further studies are needed to comprehensively explore the hidden potential and features of this enigmatic cytokine.
